# Supplementary material for: The dyskerin ribonucleoprotein complex as an OCT4/SOX2 coactivator in embryonic stem cells
Source: eLife. 2014 Nov 19;3:e03573. doi: 10.7554/eLife.03573 (PMC4270071; doi:10.7554/eLife.03573)
Supplement: Supplementary file 1. — Primer sequences. DOI: http://dx.doi.org/10.7554/eLife.03573.023 [file elife03573s001.docx]

**SUPPLEMENTARY FILE 1**

**Primer sequences**

**Figure 5**

mOct4 -2000

GGAACTGGGTGTGGGGAGGTTGTA; AGCAGATTAAGGAAGGGCTAGGACGAGAG

mOct4 -251

AGCAACTGGTTTGTGAGGTGTCCGGTGAC; TCCCCAATCCCACCCTCTAGCCTTGAC

mNanog -199

GGGTCACCTTACAGCTTCTTTTGCATTA; GGCTCAAGGCGATAGATTTAAAGGGTAG

mNanog -952

GGCAAACTTTGAACTTGGGATGTGGAAATA; CTCAGCCGTCTAAGCAATGGAAGAAGAAAT

mSox2 +3515

TTTTCGTTTTTAGGGTAAGGTACTGG; CGTGAATAATCCTATATGCATCACAAT

mSox2 -3581

CCCTGTTCCAAGTCTCTTTCTG; GATTTCAATCCAACACCATCATAG

mFgf4 -1581

ccccaaagcagtttgatgat; ccttagtctgggcactcctg

mFgf4 +2932

gggaggctacagacagcaag; ctgtgagccaccagacagaa

hNanog -190

GCTGGGTTTGTCTTCAGGTTC; CACACCCCCTACTGACCCAC

hNanog -5000

CCCTACCCCAACCTCCATTA; GGAGGCACAGTGAGACCTTG

hOct4 -2000

GGTAGATTATGGGGCCTGGT; TGTGGAGATTCCAGCCAAAT

hOct4 +1500

TCTGTTTTGGGGTTTTGGAA; TGGCTGTGTGCTCCGTTTAT

**Figure 6**

mNanog

Cctcagcctccagcagatgc; ccgcttgcacttcaTcctttg

mOct4

GAAGCAGAAGAGGATCACCTTG; TTCTTAAGGCTGAGCTGCAAG

mSox2

GAGTGGAAACTTTTGTCCGAGA; GAAGCGTGTACTTATCCTTCTTCAT

mKlf4

CAGTGGTAAGGTTTCTCGCC; GCCACCCACACTTGTGACTA

mFgf4

GGGAGGCTACAGACAGCAAG; CTGTGAGCCACCAGACAGAA

mFgf5

TTGCGACCCAGGAGCTTAAT; CTACGCCTCTTTATTGCAGCAT

mT

CTCTAATGTCCTCCCTTGTTGCC; TGCAGATTGTCTTTGGCTACTTTG

mGata6

TTGCTCCGGTAACAGCAGTG; GTGGTCGCTTGTGTAGAAGGA

mCdx2

AGGCTGAGCCATGAGGAGTA; CGAGGTCCATAATTCCACTCA

mActb

AGAGGGAAATCGTGCGTGAC; CAATAGTGATGACCTGGCCGT

**Figure 7, 8**

mDkc1

TTAGGACAACGACACCACCA; CCCAGCTGGACATAATGCTT

mEcad

GCACTCTTCTCCTGGTCCTG; GTTGACCGTCCCTTCACAGT

mEpcam

GCTGGCAACAAGTTGCTCTCTGAA; CGTTGCACTGCTTGGCTTTGAAGA

mSlug

CACATTCGAACCCACACATTGCCT; TGTGCCCTCAGGTTTGATCTGTCT

mSnail

TTGTGTCTGCACGACCTGTGGAAA; TCTTCACATCCGAGTGGGTTTGGA

mZeb1

GCTGGCAAGACAACGTGAAAG; GCCTCAGGATAAATGACGGC

mActb

GATCTGGCACCACACCTTCT; GGGGTGTTGAAGGTCTCAAA

mGapdh

TCAATGAAGGGGTCGTTGAT; CGTCCCGTAGACAAAATGGT
